# Uncoupling p38α nuclear and cytoplasmic functions and identification of two p38α phosphorylation sites on β-catenin: implications for the Wnt signaling pathway in CRC models

**DOI:** 10.1186/s13578-023-01175-4

**Published:** 2023-12-01

**Authors:** Martina Lepore Signorile, Candida Fasano, Giovanna Forte, Katia De Marco, Paola Sanese, Vittoria Disciglio, Elisabetta Di Nicola, Antonino Pantaleo, Cristiano Simone, Valentina Grossi

**Affiliations:** 1 Medical Genetics, National Institute of Gastroenterology, IRCCS “Saverio de Bellis” Research Hospital, Castellana Grotte (Ba), 70013 Bari, Italy; 2 Department of Translational and Precision Medicine, Sapienza University of Rome, 00185 Rome, Italy; 3 Medical Genetics, Department of Precision and Regenerative Medicine and Jonic Area (DiMePRe-J), University of Bari Aldo Moro, 70124 Bari, Italy

**Keywords:** p38α, Wnt/β-catenin pathway, Colorectal cancer, Chromatin-associated kinase

## Abstract

**Background:**

Activation of the Wnt pathway has been linked to colorectal cancer (CRC). Previous reports suggest that Wnt3a can activate p38. Besides, p38α feeds into the canonical Wnt/β-catenin pathway by inhibiting GSK3β through phosphorylation. Recently, we identified p38α as a new druggable member of β-catenin chromatin-associated kinase complexes in CRC.

**Methods:**

The functional relationship between p38α and β-catenin was characterized in CRC cells, patient-derived CRC stem cells, patient-derived tumor intestinal organoids, and in vivo models (C57BL/6-APC^Min/+^ mice). The role of p38α in β-catenin transcriptional activity was assessed by pharmacological inhibition with ralimetinib.

**Results:**

We used the GSK3β inhibitor TWS-119, which promotes the activation of Wnt signaling, to uncouple p38α nuclear/cytoplasmatic functions in the Wnt pathway. Upon GSK3β inhibition, nuclear p38α phosphorylates β-catenin at residues S111 and T112, allowing its binding to promoter regions of Wnt target genes and the activation of a transcriptional program implicated in cancer progression. If p38α is pharmacologically inhibited in addition to GSK3β, β-catenin is prevented from promoting target gene transcription, which is expected to impair carcinogenesis.

**Conclusions:**

p38α seems to play a dual role as a member of the β-catenin destruction complex and as a β-catenin chromatin-associated kinase in CRC. This finding may help elucidate mechanisms contributing to human colon tumor pathogenesis and devise new strategies for personalized CRC treatment.

**Supplementary Information:**

The online version contains supplementary material available at 10.1186/s13578-023-01175-4.

## Introduction

Colorectal cancer (CRC) is the main malignant tumor affecting the gastrointestinal tract and the third most common cancer in both men and women [1, 2]. CRC usually arises from adenomas, which gradually progress to carcinomas through increases in size, dysplasia, and the acquisition of villous morphology [3]. Enhanced proliferation is initially induced by the loss or inactivation of the familial adenomatous polyposis coli (*APC*) gene on chromosome 5q (90%) or by *CTNNB1* activating mutations on chromosome 3p (5%). One of the hyperproliferating cells may then give rise to a small adenoma by clonal expansion. Subsequently, the occurrence of mutations in the RAS pathway (involving *KRAS* in about 40% of CRCs or *BRAF* in about 10% of CRCs [4]) produces a larger and more dysplastic adenoma. This is usually followed by allelic deletions of chromosomes 18q and 17q with loss of *SMAD4* and *TP53*. As tumors continue to progress, the accumulated inactivation of suppressor genes correlates with the ability of carcinomas to metastasize and cause death [5, 6].

Activation of the Wnt pathway has been linked to CRC; indeed, abnormalities of chromosome 5q have been recognized as early events in the carcinogenic process leading to sporadic and hereditary CRCs [7]. In the canonical Wnt pathway, in the absence of secreted Wnt ligands (e.g., Wnt3a), APC, Axin-1, CK1, and GSK3β are assembled in the ‘destruction complex’ that traps β-catenin. Subsequently, CK1 phosphorylates β-catenin at S45. This priming phosphorylation allows GSK3β to phosphorylate β-catenin at T41, S37, and S33 [8]. S33 and S37 doubly phosphorylated β-catenin is ultimately recognized by the β-TrCP E3 ubiquitin ligase complex, ubiquitinated, and rapidly degraded by the 26S proteasome [9]. In the presence of Wnt ligands, Wnt3a binds to Frizzled and activates the G-proteins Gα_o_ and Gα_q_ as well as the downstream phosphoprotein Dishevelled, thereby inhibiting GSK3β activity. Stabilized β-catenin then translocates into the nucleus and forms complexes with members of the T cell factor (TCF)/lymphoid enhancer factor (LEF) family of transcription factors, exerting its oncogenic role by activating the transcription of many regulatory genes, such as *c-Myc* [10].

Recent advances suggest the existence of a functional relationship between the Wnt and p38 MAPK signaling pathways.

Currently, p38 is believed to act as a tumor suppressor in normal cells at the onset of cellular transformation, while it seems to function as an oncogene after the acquisition of a malignant phenotype [11, 12]. Our group has previously reported that p38α, one of the four p38 isoforms (α, β, γ, and δ), is required for CRC cell proliferation and survival, and its genetic depletion or the pharmacological blockade of its kinase activity induces growth arrest, autophagy, and cell death both in vitro and in vivo [13–15].

Various studies have suggested that p38α plays a role in chemoresistance by supporting survival or delaying ongoing cell death processes [16–18]. Indeed, it has been shown that p38α signaling is activated in cisplatin-treated CRC cells, and p38α genetic ablation or pharmacological blockade sensitizes chemoresistant cells to cisplatin [17].

p38α is relatively inactive in its non-phosphorylated form and becomes rapidly activated upon phosphorylation of two Thr-Gly-Tyr motifs [19, 20]. Phosphorylated p38α proteins can activate a variety of kinases, including MNK1, MNK2, MSK1, PRAK, MAPKAPK2, and MAPKAPK3, which are involved in the regulation of cytoplasmic and/or nuclear signaling networks and in the response to cytokines, growth factors, toxins, and pharmacological drugs [21]. Intriguingly, p38α can also be considered a prototype of chromatin-associated kinases. Indeed, it is able to associate with and phosphorylate many transcription factors and can recruit subunits of the SWI/SNF ATP-dependent remodeling complexes directly to the DNA, thereby modulating chromatin structure and transcription [22].

Recent findings identified crosstalk between the p38 and Wnt pathways. Indeed, Wnt ligands have been reported to be able to activate p38. In totipotent mouse F9 teratocarcinoma cells, Wnt3a binds to Frizzled and activates Gα_q_ and Dishevelled, which ultimately promotes transient activation of p38 [23]. Moreover, in mesenchymal cells, Wnt3a and Wnt4 activate p38 and promote osteogenic differentiation [24]. On the other hand, it seems that the p38 pathway feeds into the canonical Wnt pathway at the level of GSK3β and β-catenin. Indeed, Thornton and colleagues demonstrated that p38 can phosphorylate GSK3β at T390, thereby inactivating GSK3β kinase activity on β-catenin in thymocytes [25]. Recently, we identified p38α as a new druggable member of β-catenin chromatin-associated kinase complexes in colorectal model systems. Our data suggest that p38α serves as a regulator of gene expression by interacting with β-catenin-TCF/LEF transcriptional complexes that are recruited to Wnt-responsive elements [18].

Despite this evidence, the exact role played by p38α in the Wnt signaling pathway remains unclear. Elucidating the underlying molecular mechanisms might be beneficial for designing new therapeutic strategies to counter chemoresistance and improve treatment response in CRC patients.

## Materials and methods

### Cell lines and organoids

HCT-116 and HT-29 cells were purchased from ATCC and cultured in DMEM (11360-070, Gibco) with 10% FBS (0270-106, Gibco) and 100 IU/mL penicillin–streptomycin (15140-122, Gibco).

CRC-stem cells (CRC-SCs) were isolated from CRC patients and propagated as tumorspheres. Briefly, human colon tissue fragments were obtained in accordance with the ethical standards of the Institutional Committee on Human Experimentation after informed consent. Tumor samples were subjected to mechanical and enzymatic dissociation using the human Tumor Dissociation Kit (130-095-929, Miltenyi Biotec) according to the manufacturer’s instructions. For magnetic separation, cells were labeled with CD44 microbeads (130-095-194, Miltenyi Biotec) 24–48 h after dissociation using the Miltenyi Biotec cell isolation kit (130-108-339, Miltenyi Biotec). CRC-SCs were maintained in DMEM/F12 Advanced (12491015, Gibco) supplemented with 6 mg/mL glucose (G8769, Sigma-Aldrich), 2 mM l-glutamine (25030081, Gibco), 10 ng/mL bFGF (F0291, Sigma-Aldrich), 20 ng/mL EGF (E9644, Sigma-Aldrich), B27 supplement (12587010, Gibco), and N-2 supplement (17502-048, Gibco). Patient-derived CRC organoids were generated using IntestiCult™-SF Organoid Growth Medium (Human) (100-0340, STEMCELL Technologies) according to the manufacturer’s instructions.

All cell lines were tested to be mycoplasma-free (117048, Minerva Biolabs). All cell cultures were performed in a 37 °C and 5% CO_2_ incubator.

### RNA interference

HCT-116 cells were transfected with 50 nM validated siRNAs (Ambion) directed against (p38α) MAPK14 using HiPerfect reagent (301704, QIAGEN) according to the manufacturer’s instructions.

### In vivo studies

For in vivo studies, normal and adenocarcinoma colon mucosa tissues were obtained from C57BL/6 mice (n = 12) and from APC^Min/+^ mice (n = 24) treated with azoxymethane (AOM) (A5486, Sigma-Aldrich), respectively. Four-month-old APC^Min/+^ male mice were administered with AOM (14 mg/kg body weight) once a week for 5 weeks; one month later, they were subjected to daily intraperitoneal injections of the p38α inhibitor SB202190 (S7067, Sigma-Aldrich) (0.05 μmol/kg body weight) or DMSO for 14 days and then sacrificed. Body weight was recorded daily. Procedures involving animals and their care were conducted in conformity with the institutional guidelines that comply with national and international laws and policies.

### Histology and immunohistochemistry

Tissue specimens were fixed in 4% buffered formalin, embedded in paraffin, and sectioned at 4 mm thickness. Sections were dewaxed and rehydrated in dH_2_O. Endogenous peroxidase activity was blocked by incubation in 3% hydrogen peroxide for 10 min. Antigen retrieval was conducted in 10 mM sodium citrate buffer (pH 6.0) for 30 min. Sections were incubated overnight with anti-p38α (1:200, 9218, Cell Signaling Technologies) or anti-β-catenin (1:400, 9562, Cell Signaling Technologies) primary antibodies. Then, they were incubated with secondary biotinylated antibodies and subsequently with streptavidin–biotin-peroxidase (Envision + System HRP anti-rabbit and anti-mouse, K8002, Agilent). Samples were developed with DAB and mounted with permanent mounting media. Negative controls were used in each experiment. p38α and β-catenin immunoreactivity was evaluated through a semiquantitative approach by two independent pathologists. Ten fields with an equal area were selected for analysis at 40 × magnification. Protein expression was assessed with ImageJ software and reported as a positivity percentage.

### Chemicals

TWS-119 (S1590) and ralimetinib (S1494) were purchased from SelleckChem. Wnt3a (H17001) and SB202190 (S7067) were purchased from Sigma-Aldrich.

### Immunoblotting

Nuclear and cytoplasmic fractions were obtained by using the Nuclear Extraction Kit (ab113474, Abcam) according to the manufacturer’s instructions. Immunoblots were carried out as previously described [13]. Briefly, cells were lysed in nuclear and cytoplasmatic lysis buffer and samples were electrophoresed in a 10% SDS–polyacrylamide gel and transferred to nitrocellulose membranes (1704156, Bio-Rad Laboratories). Blots were blocked for 1 h at room temperature in blocking buffer (1X TBS, 0.1% Tween-20 with 5% w/v non-fat dry milk) and washed with TBS-T. Membranes were incubated with the indicated antibodies (Cell Signaling Technologies; 1:1000) in 10 mL of primary antibody dilution buffer (1X TBS, 0.1% Tween-20 with 5% BSA) overnight at 4 °C. Then, blots were extensively washed and incubated with horseradish peroxidase-conjugated anti-rabbit/mouse secondary antibodies in blocking buffer for 45 min, washed, and developed with the ECL-Plus Chemiluminescence Reagent (RPN2232, GE Healthcare) according to the manufacturer’s instructions.

Primary antibodies: anti-p38α MAPK (9228), anti-Lamin B1 (12586S), anti-PDI (2446S), anti-β-catenin (9562S), anti-c-Myc (9402), anti-p-β-catenin (9561), anti-p-GSK3β (35480), anti-GSK3β (9315), and anti-Vinculin (13901) all from Cell Signaling Technologies. Secondary antibodies: Rabbit IgG HRP and Mouse IgG HRP (NA934V and NA931V, respectively, GE Healthcare). Densitometric evaluation was performed using ImageJ software.

### Immunofluorescence

Cells were seeded on glass coverslips, fixed in 4% paraformaldehyde, and permeabilized using 0.01–0.1% Triton X-100. Coverslips were incubated with the indicated primary antibodies and then with Alexa Fluor 488 (A-11094, Thermo Fisher Scientific) and 647 (A-32728, Thermo Fisher Scientific) secondary antibodies; nuclei were counterstained using DAPI (D9542, Sigma-Aldrich). Slides were sealed using Vectashield Mounting Medium (H1000, Vector Laboratories). Images were acquired using a Zeiss fluorescence microscope. Primary antibodies: p38α (9228, Cell Signaling Technologies), β-catenin (8480, Cell Signaling Technologies), c-Myc (9402, Cell Signaling Technologies), and FLAG (F1804, Sigma-Aldrich).

### Quantitative real-time PCR

Total RNA was isolated using TRIzol reagent (93289, Sigma-Aldrich) according to the manufacturer’s instructions. To avoid possible DNA contamination, RNA was treated with DNase-1 (AM2224, Ambion). RNA purity was confirmed by spectrophotometry and RNA integrity by agarose gel electrophoresis. cDNA was synthesized by retro-transcribing 1 μg of total RNA using the iScript cDNA Synthesis Kit (1708890, Bio-Rad Laboratories) according to the manufacturer’s instructions. Real-time PCR primers were designed using Primer Express software. PCR assays were performed in 96-well optical reaction plates using a QuantStudio 3 machine (Thermo Fisher Scientific). Each assay was carried out in triplicate wells. Baseline values of amplification plots were set automatically and threshold values were kept constant to obtain normalized cycle times and linear regression data. The following reaction mixture per well was used: 5 μL of SYBR Green PCR Master Mix (1725240, Bio-Rad Laboratories), 1.2 μL of primers at a final concentration of 100 nM, 2.3 μL of RNAse-free water, 1.5 μL of cDNA. For all experiments, the following PCR conditions were used: denaturation at 95 °C for 30 s, followed by 40 cycles of 15 s at 95 °C and 30 s at 60 °C. Quantitative normalization of cDNA in each sample was performed using β-actin as an internal control. Relative quantification was done using the ddCT method. Primer sequences are available upon request. Results are representative of at least three independent experiments.

### Cloning and mutagenesis

The plasmids described in this manuscript were generated with specific primers, as previously described [26]. Site-directed mutagenesis was performed using the Q5® Site-Directed Mutagenesis Kit (E05545, New England Biolabs) according to the manufacturer’s instructions. The human beta-catenin pcDNA3 plasmid (16828) was purchased from Addgene. The human beta-catenin pcDNA3-S111A and human beta-catenin pcDNA3-T112A constructs were generated by site-directed mutagenesis, using the following primers:

- SDM_ beta-catenin _S111A_FW 5'-P-GCAGATCCCAGCTACACAGTT,
- SDM_ beta-catenin _S111A_RV 5'-P-ATGCCCTCATCTAATGTCTCA,
- SDM_ beta-catenin _T112A_FW 5'-P-GATCCCATCTGCACAGTTTGATG.
- SDM_ beta-catenin _T112A_ RV 5'-P- TGCATGCCCTCATCTAATG.

The pGEX-4T-3 GST-beta-catenin Wild-Type, pGEX-4T-3 GST-beta-catenin S111A, and pGEX-4T-3 GST-beta-catenin T112A constructs were generated by cloning the relevant β-catenin fragments from human beta-catenin pcDNA3, human beta-catenin pcDNA3-S111A, and human beta-catenin pcDNA3-T112A, respectively, into the pGEX-4T-3 GST Expression Vector (28-9545-52, GE Healthcare), using the BamHI and NotI enzymes.

### Recombinant protein expression/purification

BL21(DE3) Competent Cells (C2527H, Biolabs) transformed with different constructs were grown in Luria Broth medium with ampicillin (A9518, Sigma) and induced with 200 nM IPTG when they reached the optical density of 0.6 (A600) at 37 °C, overnight. Cells were then collected by centrifugation, and pellets were lysed with B-PER lysis buffer (78248, Thermo Fisher Scientific). The lysate was centrifuged at 20,000×*g* for 20 min at 4 °C. Recombinant protein expression was confirmed by SDS-PAGE. GST-Fusion proteins were purified using Pierce Glutathione Magnetic Agarose Beads (78601, Thermo Fisher Scientific) according to the manufacturer’s instructions. GST-fused proteins were evaluated and quantified by SDS-PAGE.

### Co-immunoprecipitation

Cells were collected and homogenized in lysis buffer (50 mM Tris–HCl pH 7.4, 5 mM EDTA, 250 mM NaCl, and 1% Triton X-100) supplemented with protease and phosphatase inhibitors. Coupling was performed between Dynabeads Protein A or G (10002D or 10003D, Thermo Fisher Scientific) and antibodies in 100 μL of 0.01% Tween20-1X PBS for 45 min at room temperature on a rocking platform. Cell lysates were immunoprecipitated with antibody-bead complexes. Immunocomplexes were washed extensively and boiled in Laemli sample buffer and subjected to SDS-PAGE and immunoblot analysis. Input corresponds to 10% of the lysate. IgGs were used as negative controls. Primary antibodies: p38α (9218, Cell Signaling Technologies), β-catenin (9562, Cell Signaling Technologies), GSK3β (9315, 9832 Cell Signaling Technologies), APC (2504, Cell Signaling Technologies), Axin1 (2087, Cell Signaling Technologies), FLAG-M2 (F1804, Abcam). Rabbit IgG HRP (NA934V, GE Healthcare) was used as a secondary antibody and revealed using the ECL-plus chemiluminescence reagent (RPN2232, GE Healthcare). Results are representative of at least three independent experiments.

### Mass spectrometry analysis

Mass spectrometry analysis was performed by the Cogentech SRL service. Gel bands were subjected to reduction with dithiothreitol (DTT) (A39255, Thermo Fisher Scientific), alkylation with iodoacetamide (IAA) (A39271, Thermo Fisher Scientific), and digestion with trypsin (90059, Thermo Fisher Scientific). Peptides were loaded onto a TiO_2_ resin for phospho-enrichment. Then, the flow-through was treated with C18 Spin Tips & Columns (84850, Thermo Fisher Scientific) for desalting. The samples enriched for phospho-peptides and the desalted flow-through were further purified with SP3 and then analyzed by nLC-ESI–MS/MS on a Q Exactive HF mass spectrometer (Thermo Fisher Scientific) with a 32 min gradient. Samples were run in technical duplicate, in a positive mode with electrospray ionization. Data acquisition and processing were performed with Analyst TF (version 1.7.1, AB SCIEX). Data were analyzed using the Proteome Discoverer, Mascot, and Scaffold setting software. The parameter settings of data processing were as follows: DataBase = Uniprot_CP_Human_2020_GST_CTNB1 (Database di Uniprot_cp_Human + GST CTNB1 Human sequence, Accession Number P010101); Enzyme = Trypsin (cuts at C-term of K and also on R); Modifications = Acetyl (Protein N-term), Carbamidomethyl (C), Oxidation (M), Phosphorylation (STY); Peptide Thresholds: 95.0% minimum; Protein Thresholds: 99.0% minimum; 2 peptides/protein minimum.

### Chromatin immunoprecipitation

Chromatin isolated from HT-29 and HCT-116 cells was subjected to chromatin immunoprecipitation using the MAGnify Chromatin Immunoprecipitation System (492024, Thermo Fisher Scientific) according to the manufacturer’s instructions. Briefly, cells were cross-linked in 1% formaldehyde (252549, Sigma-Aldrich) for 10 min. Cross-linking was blocked with 0.125 M glycine (23390.4, Serva) for 5 min; then, cells were washed with 1 × PBS and lysed in lysis buffer. Chromatin was sonicated to a fragment length of about 200–500 bp and immunoprecipitated with 1 µg of anti-p38α (8690, Cell Signaling Technologies), anti-β-catenin (9562S, Cell Signaling Technologies), rabbit IgGs, FLAG-M2 (F1804, Abcam), or mouse IgGs. Quantitative real-time PCR was performed using SYBR Green IQ reagent (1708880, Bio-Rad Laboratories) with the CFX Connect detection system (Bio-Rad Laboratories). Primer sequences are available upon request. Results are representative of at least three independent experiments.

### In vitro kinase activity

The analysis of p38α kinase activity was performed using the ADP-Glo Kinase Assay (V9101, Promega) according to the manufacturer’s instructions. 10 ng of p38α active protein (V2701, Promega) were assayed in a kinase reaction buffer with 500 ng of human recombinant β-catenin protein (β-catenin-WT, β-catenin-S111A, β-catenin-T112A), 150 μM ATP, and varying concentrations (0.01, 0.1, and 1 μM) of ralimetinib (S1494, SelleckChem). A total of 500 ng of p38 peptide substrate was used as a control. The generated luminescence was measured using a SPECTROstar Omega microplate reader (BMG Labtech).

### Droplet digital PCR (ddPCR) assay

ddPCR was performed using 1 ng of RNA extracted from the HCT-116 CRC cell line or 20 ng of RNA extracted from patient-derived CRC-SC tumorspheres using the Purelink RNA Micro Kit (12183016, Thermo Fisher Scientific) and retro-transcribed to cDNA using the SuperScript VILO cDNA Synthesis Kit (11755050, Thermo Fisher Scientific) according to the manufacturer’s instructions. Reactions were prepared using the ddPCR Supermix for Probes Kit (1863024, Bio-Rad Laboratories) according to the manufacturer’s instructions. 20 µL of each reaction mix were converted into droplets with the QX200 Droplet Generator (Bio-Rad Laboratories). The droplets were transferred to a 96-well plate, sealed, and cycled in a C100 Thermocycler (Bio-Rad Laboratories) under the following cycling protocol: 25 °C for 3 min, 95 °C for 10 min followed by 40 cycles of 94 °C for 30 s and 60 °C for 1 min, a post-cycling step of 98 °C for 10 min, and infinite hold at 4 °C. The plate was then transferred into a QX200 Reader (Bio-Rad Laboratories).

For patient-derived CRC organoids, 10 ng of RNA were extracted using the Purelink RNA Micro Kit (12183016, Thermo Fisher Scientific) according to the manufacturer’s instructions. The ddPCR assay was prepared using the One-Step RT-ddPCR Advanced Kit for Probes (1864021, Bio-Rad Laboratories) according to the manufacturer’s instructions. 20 µL of each reaction mix were converted into droplets with the QX200 Droplet Generator (Bio-Rad Laboratories). The droplets were transferred to a 96-well plate, sealed, and cycled in a C100 Thermocycler (Bio-Rad Laboratories) under the following cycling protocol: 25 °C for 3 min, reverse transcription at 50 °C for 60 min, 95 °C for 10 min followed by 40 cycles of 95 °C for 30 s and 55 °C for 1 min, a post-cycling step of 98 °C for 10 min, and infinite hold at 4 °C. The plate was then transferred into a QX200 Reader (Bio-Rad Laboratories).

The data were analyzed using Bio-Rad QX Manager 1.2 Standard Edition. Probes were as follows:

- c-Myc: ddPCR Gene Expression Assay, MYC, Human, Homo sapiens (dHsaCPE5051056, Bio-Rad Laboratories).
- β-catenin: ddPCR Gene Expression Assay, β-catenin, Human, Homo sapiens (dHsaCPE5040214, Bio-Rad Laboratories).

### TOPFlash/FOPFlash assay

To assess the transcriptional activity of β-catenin in HT-29 cells, we took advantage of the TOP/FOP reporter system using a dual-luciferase kit (Dual-GloTM Luciferase Assay System, E2920, Promega). On the day of the seeding, 6 × 10^5^ HT-29 cells were co-transfected with 1 µg of p38 and 1 µg of β-catenin plasmids, using the Neon transfection system (NEON, ThermoFisher Scientific) according to the manufacturer’s instructions, and plated onto 24-wells. After 24 h, cells were transiently transfected with 2 ng of Renilla luciferase vector (E2231, Promega) and 100 ng of TOPFlash β-catenin-responsive firefly luciferase reporter plasmid (17285, Millipore) or the FOPFlash negative control (17285, Millipore) using Lipofectamine 3000 (L3000001, ThermoFisher Scientific) and serum-starved for 24 h. Then, cells were stimulated with Wnt3a (50 ng/mL) and TWS-119 (10 μM) for 4 h and treated or not with ralimetinib (10 μM). After 24 h in culture, cells were harvested and both firefly and Renilla luciferase activity was measured in triplicate according to the manufacturer’s instructions. Firefly luciferase activity was normalized against Renilla luciferase activity and fold increase in TOPFlash-compared to FOPFlash-transfected cells was determined.

## Results

### p38α is a druggable member of the cytoplasmic β-catenin destruction complex in in vivo CRC models

**Fig. 1:**
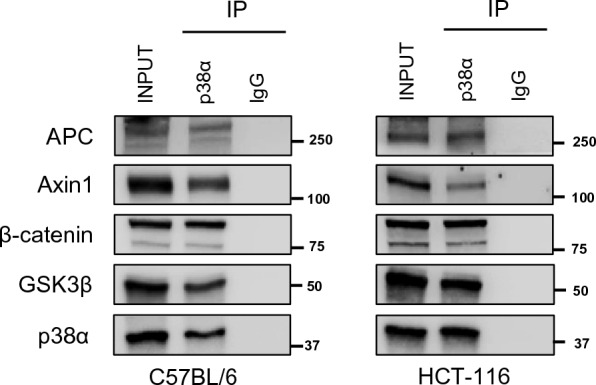
p38α is a molecular partner of the cytoplasmic β-catenin destruction complex. Co-immunoprecipitation assays showing that endogenous p38α is a molecular partner of APC, Axin1, β-catenin, and GSK3β in normal colon tissue from C57BL/6 mice and HCT-116 CRC cells. Input corresponds to 10% of the lysate. Anti-IgGs were used as negative controls. Results are representative of at least three independent experiments

Activation of p38α and nuclear β-catenin is associated with many cancers, and several genes that are regulated by these signaling pathways are crucial for cancer development and progression. Recently, we demonstrated that p38α acts as a β-catenin chromatin-associated kinase involved in tumor proliferation, metastatic dissemination, and chemoresistance [18]. In order to characterize the functional relationship between p38α and the Wnt/β-catenin pathway, here we assessed whether endogenous p38α is a partner of the multiprotein ‘destruction complex’. To this end, we analyzed the interactions between these proteins by immunoprecipitation in colon tissues derived from C57BL/6 mice and in a cellular CRC model, the HCT-116 cell line. Immunoprecipitation of whole-cell lysates with an antiserum against p38α, followed by immunoblotting, indicated that p38α is a molecular partner of APC, Axin-1, β-catenin, and GSK3β in both normal colon and CRC cells (Fig. 1).

In order to confirm the results of Thornton and colleagues [25] in our CRC model, we investigated the effect of p38α pharmacological inhibition (with the selective inhibitor ralimetinib) or genetic ablation (with a specific siRNA) on the regulation of the GSK3β phospho-inhibition signal (p-GSK3β T390) (Additional file 1: Fig. S1A, B). Immunoblot experiments showed that treatment of CRC cells with ralimetinib (Additional file 1: Fig. S1A) or a specific siRNA (Additional file 1: Fig. S1B) decreases the phosphorylation inhibitory signal of GSK3β while increasing the phospho-degradation signal of β-catenin (p-β-catenin S33/37/T41).

**Fig. 2:**
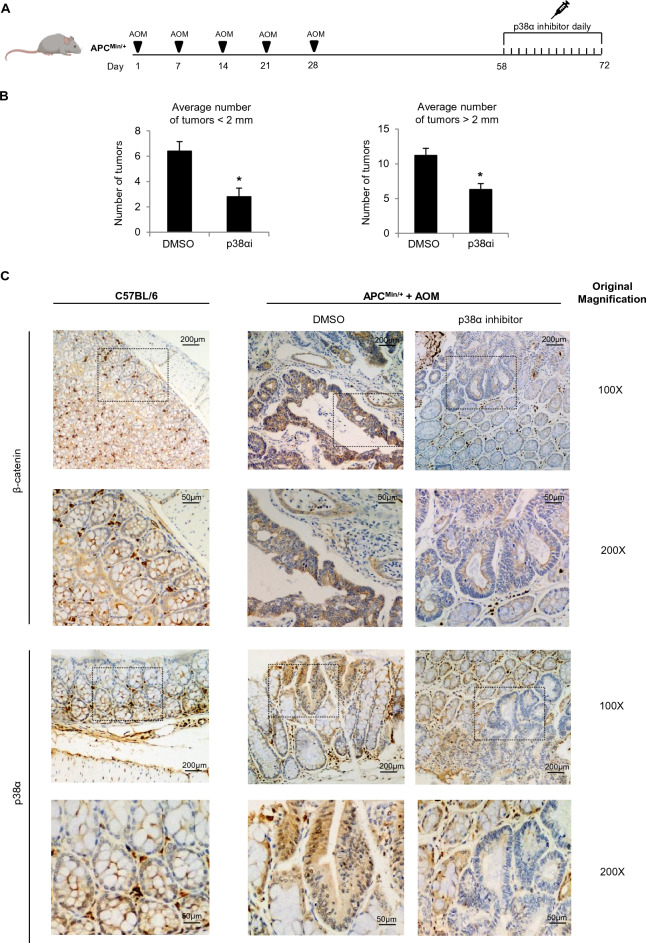
Effect of p38α inhibition in a CRC mouse model. **A** Mice treatment scheme. APC^Min/+^ mice were administered with AOM (14 mg/kg body weight) once a week for 5 weeks; one month later, they were subjected to daily intraperitoneal injections of the p38α inhibitor SB202190 (0.05 μmol/kg body weight) or DMSO for 14 days and then sacrificed. **B** Graph showing the reduction in the number of colon tumors in animals treated with SB202190. Tumors were stained with methylene blue, counted, and measured. Statistical analysis was performed using Student’s t-test: *P < 0.05 vs. DMSO. **C** Immunohistochemical analysis of p38α and β-catenin cytoplasmic and nuclear staining in C57BL/6 mice and AOM-treated APC^Min/+^ mice injected with the p38α inhibitor SB202190 or DMSO. Original magnification: 100 × and 200 × . p38αi = p38α inhibitor. Results are representative of at least three independent experiments

To get further insight into the functional relationship between p38α and the Wnt/β-catenin pathway, we analyzed tumor tissues from APC^Min/+^ mice. This murine model, identified in 1990 in an ethylnitrosourea mutagenesis screen in C57BL/6 mice, represents a very useful tool to study CRC. The genetic basis for the intestinal phenotype is a T-to-A transversion at nucleotide 2549 of the mouse *APC* gene resulting in the truncation of the APC protein at amino acid 850. For this reason, the Wnt/β-catenin pathway is overactive in these mice, which develop multiple intestinal neoplasia (Min) after spontaneously losing the heterozygous wild-type *APC* allele in intestinal epithelial cells [27]. Four-month-old APC^Min/+^ mice were administered with a potent carcinogen, AOM (14 mg/kg body weight), once a week for 5 weeks to induce epithelial cell transformation from aberrant crypt foci to adenoma and malignant adenocarcinoma. One month later, animals were subjected to daily intraperitoneal injections of the p38α inhibitor SB202190 (0.05 μmol/kg body weight) or dimethyl sulfoxide (DMSO) for 14 days and then sacrificed (Fig. 2A). Macroscopic examination of the bowel showed a significant reduction in the number of tumors (both for tumors < 2 mm and > 2 mm) in the colon of p38α inhibitor-treated APC^Min/+^ mice compared with controls (Fig. 2B).

Immunohistochemical analysis of healthy colon sections from C57BL/6 mice revealed weak nuclear staining for p38α and β-catenin. Conversely, colon sections from AOM-treated APC^Min/+^ mice injected with the vehicle showed high nuclear positivity for p38α and β-catenin, whereas animals injected with the p38α inhibitor displayed decreased p38α and β-catenin nuclear staining in colonic epithelial cells (Fig. 2C; Additional file 1: Fig. S2).

These data suggest that when the Wnt/β-catenin pathway is overactive, as is the case in the APC^Min/+^ murine model, p38α and β-catenin are phospho-activated and translocate together into the nucleus. In contrast, upon p38α pharmacological inhibition, GSK3β is no longer inhibited by p38α in the cytoplasm and can phosphorylate β-catenin, targeting it for proteasome degradation.

p38α thus seems to play a dual role in the Wnt signaling pathway: the first in the cytoplasm, where it phosphorylates GSK3β, thereby inactivating its kinase activity and thus promoting β-catenin nuclear translocation, as shown in thymocytes by Thornton and colleagues [25] and confirmed in our CRC model (Additional file 1: Fig. S1A, B); and the second in the nucleus, where it supports β-catenin transcriptional activity directly on chromatin [18].

### Uncoupling p38α cytoplasmic and nuclear functions in the Wnt pathway

In this scenario, we planned to investigate the role of p38α by uncoupling its nuclear and cytoplasmic functions. To this end, we used TWS-119, a well-established inhibitor of GSK3β kinase activity, and the small-molecule p38α inhibitor ralimetinib in in vitro CRC models. Of note, ralimetinib is being evaluated in various clinical trials for inflammatory disease and cancer [28, 29]. TWS-119 allowed us to mimic the inhibitory activity of active p38α on GSK3β in the cytoplasm in culture conditions where p38α was pharmacologically inhibited with ralimetinib. In this way, we were able to counter the effect of p38α inhibition on β-catenin in the cytoplasm and assess the effect of p38α inhibition solely in the nucleus.

Stabilization of β-catenin resulting from mutations in the APC protein or β-catenin itself is observed in most CRC cell lines. The HT-29 CRC cell line is widely used for experimental studies because it retains many biochemical and physiological features of normal colorectal epithelial cells. HT-29 cells contain two carboxyl-terminal-truncated APC proteins of approximately 100 kDa and 200 kDa. Remarkably, these truncated proteins still retain at least partial activity for binding β-catenin [30]. On the other hand, HCT-116 CRC cells harbor a heterozygous mutation in the β-catenin gene and thus carry a wild-type allele and a mutant allele with deletion of S45, which is required for GSK3β-mediated β-catenin regulation [31].

**Fig. 3:**
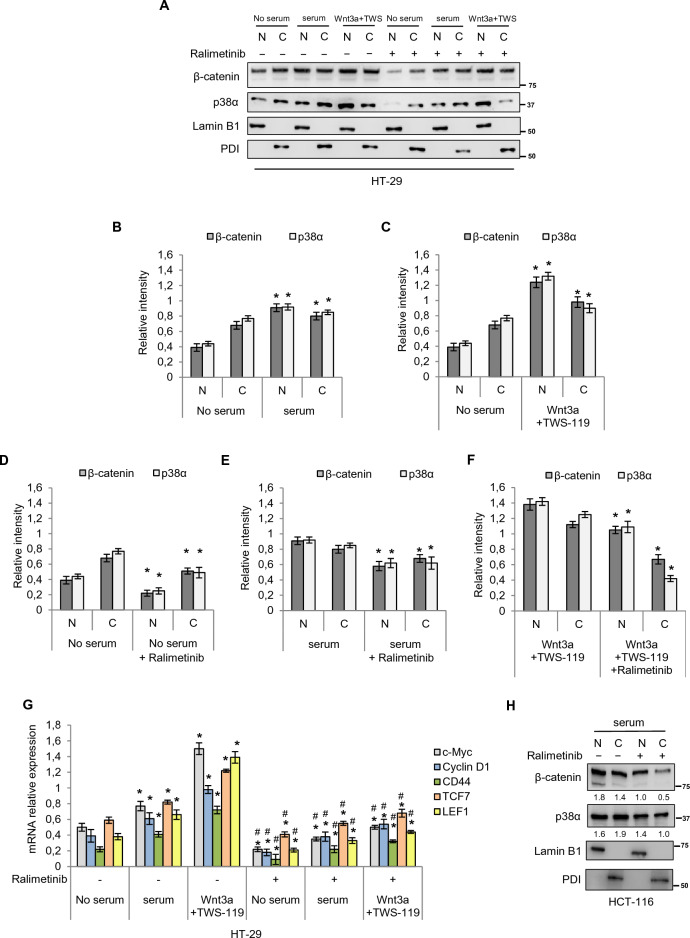
Uncoupling p38α cytoplasmic and nuclear functions in the Wnt pathway. **A**, **H** Immunoblotting analysis of p38α and β-catenin cellular localization in HT-29 (**A**) and HCT-116 (**H**) CRC cells under serum starvation (24 h) and upon activation of the Wnt pathway mediated by serum supplementation (**A**, **H**) or addition of Wnt3a (50 ng/mL) and the GSK3β inhibitor TWS-119 (10 μM) for 4 h (**A**). Subsequently, cells were treated or not with the p38α inhibitor ralimetinib (10 μM) for 24 h (**A**, **H**). **B**–**F** Densitometric analysis of the indicated protein levels against the loading control in the different culture conditions used in this study. Statistical analysis was performed using Student’s t-test: *P < 0.05 vs. no serum (**B**, **C**) or vs. no ralimetinib (**D**–**F**). **G** RTqPCR analysis of β-catenin target gene expression in HT-29 cells treated as in **A**. Statistical analysis was performed using Student’s t-test: *P < 0.05 vs. no serum; ^#^P < 0.05 vs. no ralimetinib. Lamin B1: nuclear loading control; PDI: cytoplasmic loading control. N = Nucleus, C = Cytoplasm. Results are representative of at least three independent experiments

We first evaluated p38α and β-catenin protein localization after activation of the Wnt pathway (mediated by serum supplementation or addition of Wnt3a and TWS-119), followed by inhibition of p38α with ralimetinib, in HT-29 CRC cell lines. HT-29 cells were serum-starved to retain β-catenin in the cytoplasm and then switched to a serum-supplemented medium or a medium containing Wnt3a and TWS-119. Immunoblot analysis showed that β-catenin and p38α expression was barely detectable in the nucleus under serum starvation, while it increased substantially after serum supplementation (Fig. 3A, B). Interestingly, p38α displayed the same nuclear/cytoplasmic localization of β-catenin after treatment with Wnt3a and TWS-119, with both proteins showing significant translocation into the nucleus (Fig. 3A, C). Upon serum starvation and p38α inhibition, the expression of p38α and β-catenin was strongly reduced in the nuclear fraction (Fig. 3A, D). Furthermore, the use of ralimetinib in HT-29 cells cultured in serum-containing medium released GSK3β from p38α-mediated inhibition, thus reducing the amount of p38α and β-catenin capable of translocating into the nucleus (Fig. 3A, E). Interestingly, upon activation of the Wnt pathway by addition of Wnt3a and TWS-119, followed by inhibition of p38α with ralimetinib, GSK3β activity was inhibited and β-catenin could translocate into the nucleus with p38α (Fig. 3A, F).

Thus, after activation of the Wnt/β-catenin pathway by serum supplementation or addition of Wnt3a and TWS-119, p38α translocates with β-catenin into the nucleus, where they are both active and promote the transcription of several β-catenin target genes (Fig. 3G). However, our data indicate that upon p38α pharmacological inhibition with ralimetinib, although p38α and β-catenin can still translocate into the nucleus (due to concomitant inhibition of GSK3β by TWS-119), they are prevented from fully activating the Wnt transcriptional program, as shown by the analysis of β-catenin target gene expression levels (Fig. 3G).

Furthermore, in HCT-116 cells, where β-catenin is heterozygously mutated in a residue that is required for GSK3β kinase activity (S45) [31], the addition of the GSK3β inhibitor TWS-119 was not necessary to uncouple the nuclear and cytoplasmatic functions of p38α. In these cells, β-catenin is only partially regulated by GSK3β activity and translocates into the nucleus with p38α under serum-containing culture conditions. The addition of ralimetinib, which releases GSK3β from p38α-mediated inhibition and thus allows it to phosphorylate existing wild-type β-catenin, induced a reduction in β-catenin protein levels (Fig. 3H). Nevertheless, β-catenin could still translocate into the nucleus with p38α.

**Fig. 4:**
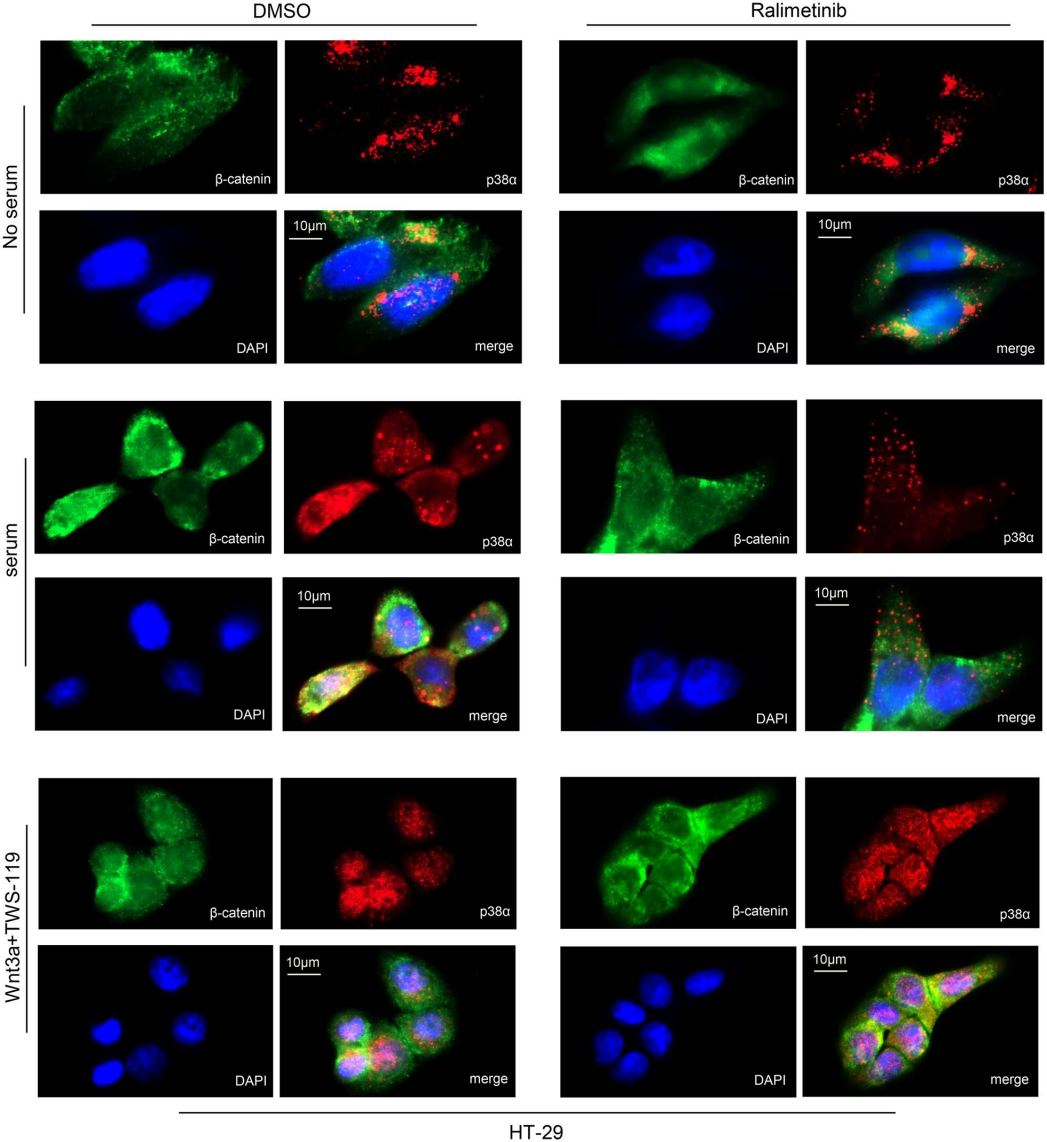
Immunofluorescence analysis of p38α and β-catenin cellular localization. Immunofluorescence analysis of p38α and β-catenin cellular localization in HT-29 CRC cells under serum starvation (24 h) and upon activation of the Wnt pathway by serum supplementation or addition of Wnt3a (50 ng/mL) and the GSK3β inhibitor TWS-119 (10 μM) for 4 h. Subsequently, cells were treated or not with the p38α inhibitor ralimetinib (10 μM) for 24 h. Results are representative of at least three independent experiments

These data were further confirmed by immunofluorescence staining (Fig. 4; Additional file 1: Fig. S3). HT-29 CRC cells were serum-starved and then switched to a serum-supplemented medium or a medium containing Wnt3a and TWS-119. Subsequently, p38α was pharmacologically inhibited with ralimetinib. Cells were stained with specific antibodies to detect p38α and β-catenin protein localization in these different culture conditions. The results gathered from these experiments corroborated the data obtained by immunoblot (Fig. 3A). In particular, we confirmed at the single-cell level that, upon addition of Wnt3a and TWS-119, p38α and β-catenin can still translocate into the nucleus in the presence of ralimetinib (Fig. 4; Additional file 1: Fig. S3).

### p38α and β-catenin synergize to activate the expression of Wnt target genes

**Fig. 5:**
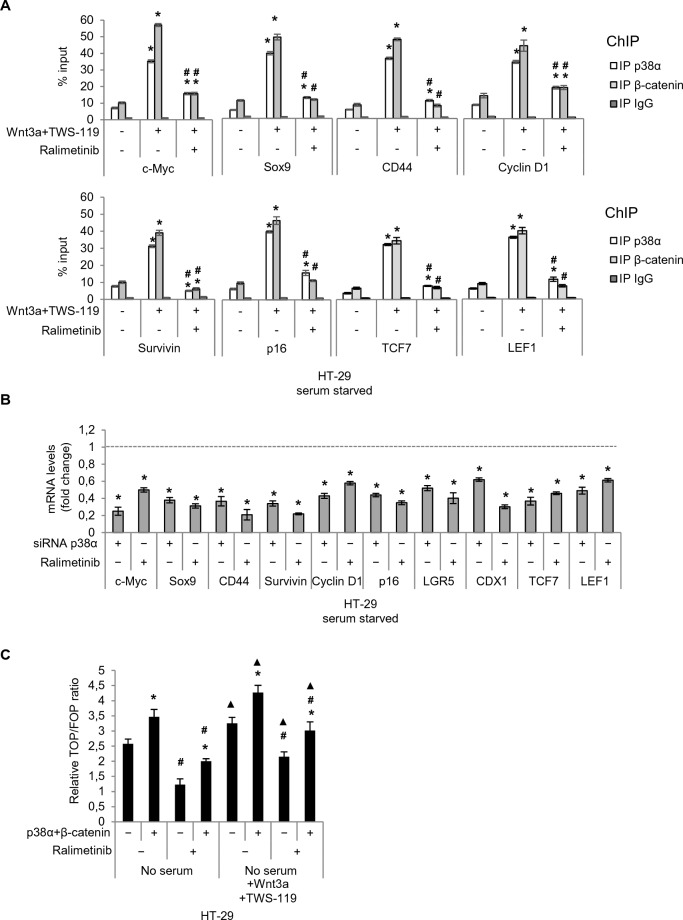
p38α modulate β-catenin target gene expression. **A** Chromatin immunoprecipitation assays of Wnt target genes in HT-29 cells under serum starvation (24 h) and upon activation of the Wnt pathway mediated by the addition of Wnt3a (50 ng/mL) and the GSK3β inhibitor TWS-119 (10 μM) for 4 h. Subsequently, cells were treated or not with the p38α inhibitor ralimetinib (10 μM) for 24 h. Quantification was done using the % input method. Statistical analysis was performed using Student’s t-test: *P < 0.05 vs. untreated cells, and ^#^P < 0.05 vs. no ralimetinib. **B** RTqPCR analysis of Wnt target genes in HT-29 cells upon activation of the Wnt pathway mediated by the addition of Wnt3a (50 ng/mL) and the GSK3β inhibitor TWS-119 (10 μM) for 4 h after p38α genetic ablation for 24 h or as a pre-treatment before p38α inhibition with ralimetinib (10 μM) for 24 h. Data are presented as mRNA fold change vs. control. The dotted line corresponds to the expression levels detected in control conditions (siRNA CTRL/DMSO). Statistical analysis was performed using Student’s t-test: *P < 0.05 vs. siRNA CTRL/DMSO. **C** TOPFlash/FOPFlash assay for Wnt transcriptional activity. HT-29 cells were first transfected to overexpress p38α and β-catenin; after 24 h, cells were transfected with TOP/FOP plasmids, serum-starved for 24 h and then stimulated with Wnt3a (50 ng/mL) and TWS-119 (10 μM) for 4 h. Subsequently, cells were treated or not with ralimetinib (10 μM) for 24 h. Statistical analysis was performed using Student’s t-test: *P < 0.05 vs. empty vector, ^#^P < 0.05 vs. DMSO, ^▲^P < 0.05 vs. no serum. Results are representative of at least three independent experiments

In order to specifically investigate the role of nuclear p38α in the regulation of β-catenin transcriptional activity, we performed a set of chromatin immunoprecipitation assays. These experiments were designed to analyze the co-occupancy of different Wnt target gene promoters by p38α and β-catenin upon p38α pharmacological inhibition. HT-29 cells were serum-starved and then switched to a culture medium containing Wnt3a and TWS-119 for 4 h. Subsequently, cells were treated with ralimetinib for 24 h. Interestingly, p38α-inhibited HT-29 cells showed significantly reduced co-occupancy of Wnt target gene promoters by p38α and β-catenin. Indeed, inhibition of p38α seems to reduce β-catenin affinity for the promoter regions of these genes (Fig. 5A). Interestingly, our results revealed that ralimetinib reduces β-catenin binding to the promoters of TCF7 and LEF1, which are considered the main transcription factors involved in the activation of the Wnt signaling pathway (Fig. 5A). β-catenin contains a potent transcriptional activation domain but requires interaction with TCF7 or LEF1 to bind to the DNA. Thus, β-catenin must first form a complex with TCF7 or LEF1 for this complex to be able to occupy Wnt-responsive elements [32]. This finding led us to hypothesize that p38α inhibition may induce a medium-term effect mediated by a negative feedback loop. Indeed, treatment with ralimetinib or p38α genetic ablation decreases the amount of TCF7 and LEF1 available in CRC cells and thus reduces the capacity of these cells to express Wnt/β-catenin target genes, including *TCF7* and *LEF1* themselves (Fig. 5B). Consistent with these data, RT-qPCR screening assays performed on Wnt target genes showed that their high mRNA expression levels detected after activation of the Wnt pathway (Wnt3a and TWS-119) decreased significantly upon p38α pharmacological inhibition with ralimetinib or genetic ablation with a specific siRNA (Fig. 5B), indicating that p38α inhibition or genetic ablation reduces β-catenin transcriptional activity in HT-29 CRC cells. To further evaluate Wnt-specific transcriptional activity, we used the TOPFlash/FOPFlash luciferase reporter system in HT-29 cells (Fig. 5C). Dual luciferase reporter assays showed increased firefly luciferase activity in cells transfected with TOPFlash (vector with intact TCF binding sites) compared to cells transfected with FOPFlash (vector with mutated TCF binding sites). Importantly, treatment with ralimetinib decreased firefly luciferase activity in cells transfected with TOPFlash, confirming that p38α inhibition reduces β-catenin transcriptional activity in these cells (Fig. 5C).

### Characterization of the mechanism of action of p38α on β-catenin

**Fig. 6:**
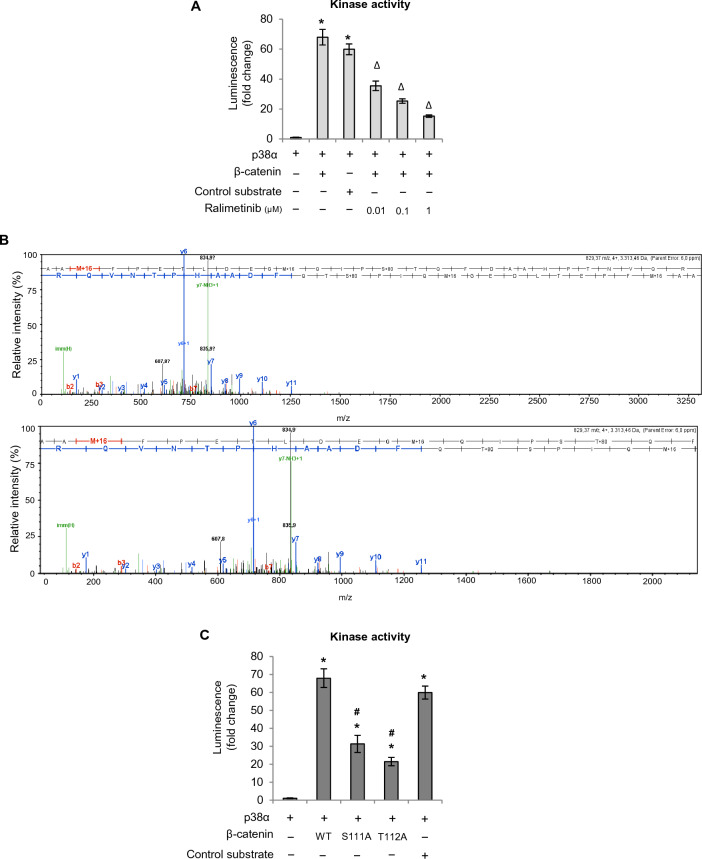
Characterization of p38α kinase activity on β-catenin. **A** In vitro kinase assay showing β-catenin phosphorylation by p38α in the absence or presence of ralimetinib at the indicated concentrations. Statistical analysis was performed using Student’s t-test: *P < 0.05 vs. active p38α; ^Δ^P < 0.05 vs. active p38α + β-catenin. **B** MS/MS spectrum of the double-charged precursor ion of peptide A96AMFPETLDEGMQIPS111T112QFDAAHPTNVQR124. **C** In vitro kinase assay showing phosphorylation of β-catenin-WT, β-catenin-S111A, and β-catenin-T112A by p38α. Results are representative of at least three independent experiments

Recently, we showed that active p38α can efficiently phosphorylate β-catenin [18]. Here we found that inhibition of p38α activity by different concentrations of ralimetinib (0.01, 0.1, and 1 μM) significantly reduced β-catenin phosphorylation by p38α in vitro (Fig. 6A). Moreover, we performed mass spectrometry (MS) analysis of β-catenin after phosphorylation by p38α in vitro. The peptide A96AMFPETLDEGMQIPS111T112QFDAAHPTNVQR124, obtained by proteolytic digestion of β-catenin with trypsin, showed a mass increase of 80 Da (which corresponds to the weight of an additional phosphate) for two consecutive amino acids, S111 and T112 (Fig. 6B). To test whether these residues were involved in β-catenin transcriptional activity, we generated two pGEX4T3-β-catenin-FLAG vectors encoding β-catenin-S111A and β-catenin-T112A and found that phosphorylation of the recombinant protein products by p38α is impaired in vitro, as shown in Fig. 6C. In particular, our results suggest that T112 is the most prominent p38α phosphorylation site on β-catenin in vitro and that S111 is also involved in this process, although to a lesser extent (Fig. 6C).

**Fig. 7:**
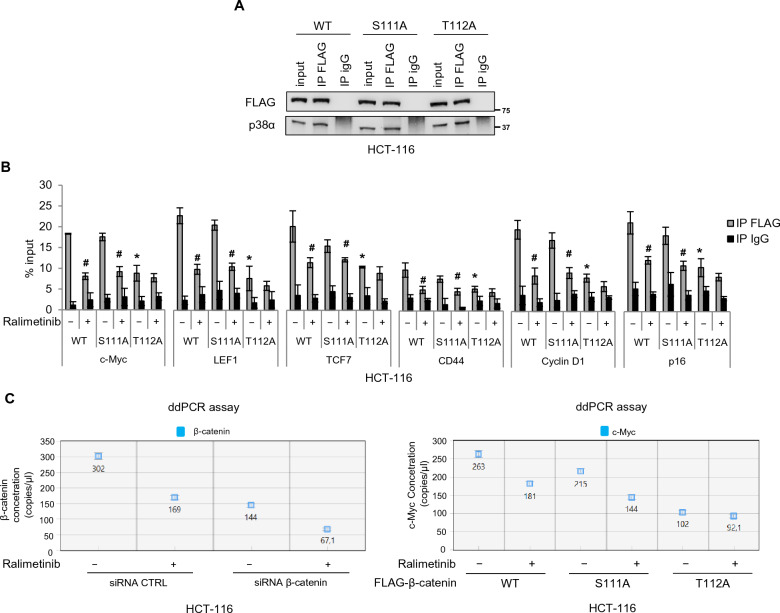
The identified β-catenin residues targeted for phosphorylation by p38α are crucial for β-catenin transcriptional activity. **A** Co-immunoprecipitation of p38α with FLAG-β-catenin-WT or FLAG-β-catenin-S111A or FLAG-β-catenin-T112A in HCT-116 cells. Input corresponds to 10% of the lysate. Anti-IgGs were used as negative controls. **B** Chromatin immunoprecipitation assay in HCT-116 cells. Cells overexpressing FLAG-β-catenin-WT or FLAG-β-catenin-S111A or FLAG-β-catenin-T112A were treated or not with ralimetinib (10 μM) for 24 h. Chromatin was pulled down with anti-FLAG antibodies. Anti-IgGs were used as negative controls. *P < 0.05 vs. FLAG-β-catenin-WT; ^#^P < 0.05 vs. DMSO. **C** Quantification results of the ddPCR assay (copies/µL) of *β-catenin* and *c-Myc* mRNA expression, as processed by QuantaSoft. HCT-116 CRC cells silenced by genetic ablation for endogenous β-catenin and exogenously expressing β-catenin-WT, β-catenin-S111A, β-catenin-T112A were treated or not with the p38α inhibitor ralimetinib (10 μM) for 24 h. The error bars represent the maximum and minimum Poisson distribution for the 95% confidence interval generated by QuantaSoft. Results are representative of at least three independent experiments

Furthermore, we carried out co-immunoprecipitation studies to investigate whether these residues (S111 and T112) are required for β-catenin and p38α physical interaction. Our results showed that this interaction was not affected in HCT-116 cells overexpressing FLAG-β-catenin-S111A or FLAG-β-catenin-T112A as compared to FLAG-β-catenin-WT-transfected cells (Fig. 7A). Subsequently, to assess the functional role of these two residues, we evaluated FLAG-β-catenin-S111A or FLAG-β-catenin-T112A occupancy of various β-catenin target gene promoters compared to FLAG-β-catenin-WT by ChIP assays. HCT-116 cells were transfected with FLAG-tagged plasmids encoding wild-type or mutant β-catenin and then immunoprecipitated with FLAG antibodies. Our results showed a reduction in mutant β-catenin occupancy of the promoter region of β-catenin target genes. This effect was more prominent for the β-catenin-T112A mutant, thereby confirming the in vitro data that T112 phosphorylation plays a more important role than S111 phosphorylation in the regulation of β-catenin transcriptional activity by p38α (Fig. 7B). As a further confirmation, p38α inhibition with ralimetinib decreased FLAG-β-catenin-WT and FLAG-β-catenin-S111A occupancy of β-catenin target genes promoters, while not showing a significant effect for FLAG-β-catenin-T112A (Fig. 7B). Then, in order to better characterize the relevance of β-catenin phosphorylation by p38α on β-catenin transcriptional activity, we performed a droplet digital PCR (ddPCR) analysis in HCT-116 cells. We first silenced β-catenin endogenous expression by genetic ablation with a specific siRNA (Fig. 7C left panel), and then exogenously expressed β-catenin-WT, β-catenin-S111A, or β-catenin-T112A. Subsequently, cells were treated with the p38α inhibitor ralimetinib for 24 h. ddPCR assays showed that genetic ablation of β-catenin induced a significant decrease in the expression of *c-Myc*, the main Wnt target gene (Additional file 1: Fig. S4A). In addition, this analysis revealed that CRC cells exogenously expressing β-catenin-WT recovered the ability to express *c-Myc*, while CRC cells expressing exogenous β-catenin-S111A or β-catenin-T112A were unable to restore *c-Myc* expression (Fig. 7C right panel). These data confirmed that phosphorylation of these two β-catenin residues by p38α is involved in the regulation of β-catenin transcriptional activity. Consistent with the in vitro kinase assay data and in cellulo ChIP data, it seems that T112 phosphorylation plays a more important role than S111 phosphorylation in this process (Fig. 7C). Moreover, in each condition, pharmacological inhibition of p38α downregulates the expression of *c-Myc* (Fig. 7C; Additional file 1: Fig. S4A).

**Fig. 8:**
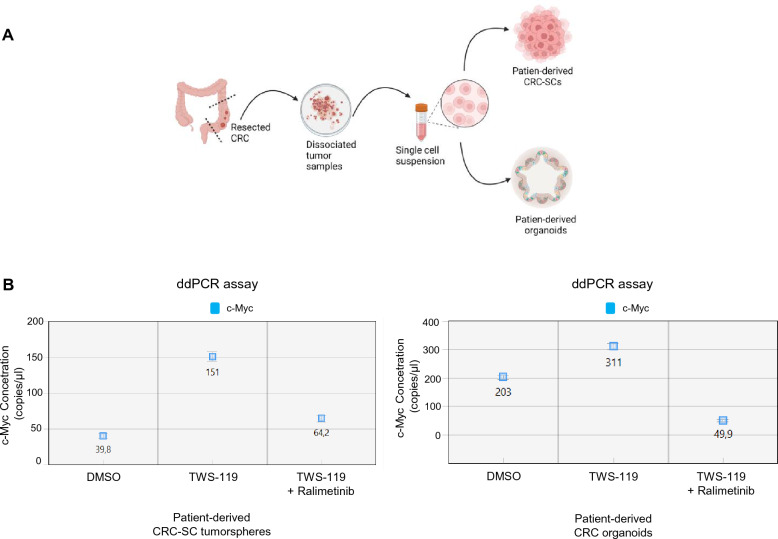
Pharmacological targeting of p38α inhibits β-catenin transcriptional activity in patient-derived CRC-SCs and tumor intestinal organoids. **A** Schematic representation of the experimental procedure used for generating patient-derived CRC-SCs and organoids (created with BioRender.com). **B** Quantification results of the digital droplet PCR (ddPCR) assay (copies/µL) of *c-Myc* mRNA expression, as processed by QuantaSoft. Patient-derived CRC-SC tumorspheres (left panel) and patient-derived CRC organoids (right panel) were treated with the GSK3β inhibitor TWS-119 (10 μM) for 4 h and subsequently treated or not with the p38α inhibitor ralimetinib (10 μM) for 24 h. The error bars represent the maximum and minimum Poisson distribution for the 95% confidence interval generated by QuantaSoft. Results are representative of at least three independent experiments

In order to increase the translational significance of our results, we also evaluated the impact of p38α pharmacological inhibition on *c-Myc* expression in patients-derived CRC-SCs and patient-derived tumor intestinal organoids (Fig. 8A). Consistent with the data described above, evaluation of *c-Myc* expression by ddPCR showed that the high mRNA levels detected after activation of the Wnt pathway (TWS-119) decreased significantly upon treatment with ralimetinib (Fig. 8B), indicating that p38α inhibition reduces β-catenin transcriptional activity also in these complex CRC cell systems.

**Fig. 9:**
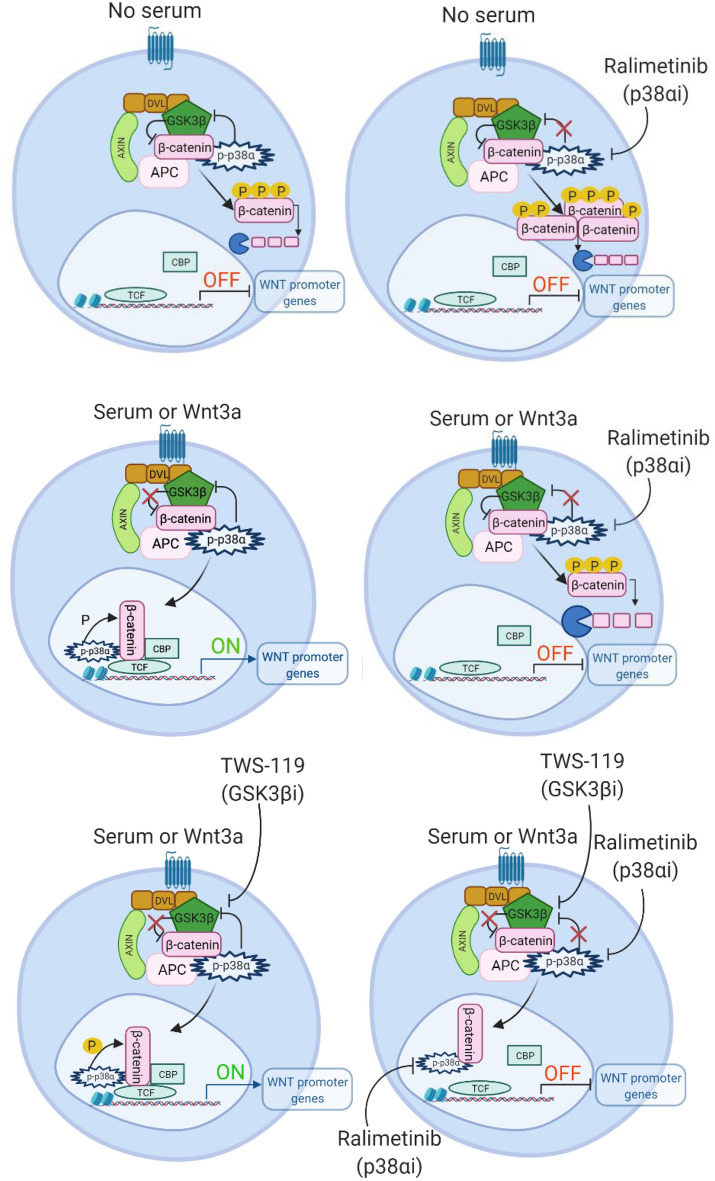
Schematic representation of p38α and β-catenin localization and activity under the different experimental conditions used in this study. *GSK3βi* GSK3β inhibitor, *p38αi* p38α inhibitor

Collectively, these data confirm that p38α acts at two different levels in Wnt signaling: the first in the cytoplasm, where it inactivates GSK3β kinase activity, thereby promoting β-catenin translocation into the nucleus [25]; and the second in the nucleus, where it sustains β-catenin transcriptional activity on chromatin [18]. Pharmacological inhibition of p38α would thus impair both of these functions, inducing the downregulation of β-catenin target gene expression (Fig. 9).

## Discussion

CRC is the second most lethal and the third most prevalent cancer in the world [33]. Due to risk factors characterizing westernization, the prevalence of CRC is increasing in high- and middle-income nations, where it represents a serious public health concern. In addition, the incidence of early-onset CRC is on the rise [33]. The major signaling cascade driving CRC tumorigenesis is the Wnt/β-catenin pathway. In fact, besides playing a crucial role in normal cell development, differentiation, and tissue homeostasis [34], this pathway is also involved in abnormal intestinal cell proliferation and tumor progression [35, 36]. Of note, recent studies showed that CRC cell proliferation and survival also rely on p38α [18, 21, 37]. Indeed, several malignancies are linked to p38α and nuclear β-catenin activation, and the genes regulated by these proteins are critical for tumor initiation and progression [18, 21, 37].

Emerging evidence indicates the existence of a functional relationship between p38 MAPK and the Wnt signaling pathway. Specifically, Thornton and colleagues investigated the cytoplasmic activity of p38α, revealing its ability to inhibit GSK3β by phosphorylation [25]. Moreover, we recently demonstrated that nuclear p38α acts as a β-catenin chromatin-associated kinase involved in tumor proliferation, metastatic dissemination, and chemoresistance [18]. Here, we uncoupled the nuclear and cytoplasmic functions of p38α in the Wnt signaling pathway by using the well-characterized GSK3β inhibitor TWS-119 and the p38α pharmacological inhibitor ralimetinib. Our findings revealed that p38α is part of the cytoplasmic β-catenin destruction complex and can translocate into the nucleus together with β-catenin in different CRC models. Indeed, after activation of the Wnt/β-catenin pathway, p38α moves with β-catenin into the nucleus, where they can both be recruited to the promoter regions of several β-catenin target genes (including *c-Myc*) and elicit transcriptional activation. Like other transcription factors, β-catenin functions are tightly regulated by protein phosphorylation and dephosphorylation [38]. Here, we found that β-catenin transcriptional activity is modulated by p38α kinase activity. In particular, we showed that p38α binds to β-catenin and phosphorylates it at T112 and S111, with T112 appearing to play a more significant role in the transcriptional activation of β-catenin target genes. Our results led us to speculate that β-catenin phosphorylation by p38α at S111 and T112 is required to stabilize β-catenin binding to target gene promoters on chromatin, thereby promoting β-catenin transcriptional activity.

T112 is a key phosphorylation site in β-catenin N-terminus and is specifically targeted by 3-phosphoinositide-dependent protein kinase 1 (PRKD1) and casein kinase II (CKII) [39, 40]. CKII-mediated phosphorylation of β-catenin at T112 is known to regulate β-catenin cytoplasmic stability, and this mechanism synergizes with GSK3β phosphorylation activity in the multi-protein complex that controls β-catenin degradation [40]. Furthermore, Du and colleagues demonstrated that PRKD1 interacts with and phosphorylates β-catenin at T112 and T120 both in vitro and in vivo. In particular, mutation of these threonine residues increased β-catenin nuclear localization and altered its transcriptional activity on *cyclin D1* and *c-Myc* genes [39]. In addition, upon phosphorylation of T112 by PRKD1, β-catenin becomes less stable and more prone to proteasomal degradation [39]. In CRC cells treated with ralimetinib, p38α and β-catenin were still able to translocate into the nucleus but they could not bind to Wnt target gene promoters. As a result, p38α-β-catenin transcriptional activity was inhibited. Similarly, inhibition of *c-Myc* was observed in patient-derived CRC-SCs and tumor intestinal organoids upon treatment with ralimetinib.

## Conclusions

This study uncouples p38α cytoplasmic and nuclear functions in the Wnt signaling pathway in CRC cells. Our results identified two β-catenin residues that are phosphorylated by p38α and seem to play an important role in β-catenin transcriptional activation. Considering the role of β-catenin activation in CRC tumorigenesis, our data suggest that p38α pharmacological inhibition is a promising approach to develop new therapeutic strategies for colorectal carcinogenesis.

The translational relevance of these findings is further strengthened by the fact that ralimetinib proved effective in CRC model systems such as patient-derived CRC-SCs and tumor intestinal organoids. Of note, ralimetinib displayed a tolerable safety profile, with evidence of antineoplastic activity, in a recent phase I clinical trial in patients with advanced or metastatic cancer (ID NCT01393990). Moreover, it has already been tested in combination with other agents in a phase I study in CRC patients (ID NCT02860780).

Taken together, the data presented in this paper may help design prospective clinical trials of tailored interventions for CRC based on p38α inhibition.

### Supplementary Information

**Additional file 1: Figure S1**. p38α pharmacological inhibition or genetic ablation modulates p-GSK3-β levels. (A-B) Immunoblotting analysis of p-β-catenin (S33/37/T41) (phospho-degradation signal) and p-GSK3β (T390) (phospho-inhibition signal) in HCT-116 CRC cells treated or not with the p38α inhibitor ralimetinib (10 μM) for 48 h (A) or silenced by genetic ablation for p38α (B). CTRL = Control. Results are representative of at least three independent experiments. **Figure S2**. Immunohistochemistry quantification of p38α and β-catenin protein expression in a CRC mouse model. Immunohistochemistry quantification of p38α and β-catenin protein expression in colon tissue from C57BL/6 and AOM-APC^Min/+^ mice treated as indicated in Fig. 2. Statistical analysis was performed using Student’s t-test: *P < 0.05 vs C57BL/6; ^#^P < 0.05 vs. DMSO. **Figure S3**. Quantification of p38α and β-catenin immunofluorescence data. Quantification of the immunofluorescence analysis of p38α and β-catenin co-staining experiments shown in Fig. 4. Statistical analysis was performed using Student’s t-test: *P < 0.05 vs No serum; ^#^P < 0.05 vs. DMSO. **Figure S4**. Analysis of β-catenin genetic ablation efficiency. (A) Quantification results of the digital droplet PCR (ddPCR) assay (copies/µL) of *c-Myc* mRNA expression as processed by QuantaSoft. HCT-116 CRC cells were silenced by genetic ablation for β-catenin and then treated or not with the p38α inhibitor ralimetinib (10 μM) for 24 h. The error bars represent the maximum and minimum Poisson distribution for the 95% confidence interval generated by QuantaSoft. (B) Immunoblotting analysis of c-Myc protein amount in HCT-116 CRC cells treated as in A. β-actin was used as a loading control. Results are representative of at least three independent experiments.

## Data Availability

All data generated or analyzed during this study are included in this manuscript and in the Additional files.
