# Supplementary figures and images for: Uncoupling p38α nuclear and cytoplasmic functions and identification of two p38α phosphorylation sites on β-catenin: implications for the Wnt signaling pathway in CRC models
Source: Cell Biosci. 2023 Dec 1;13:223. doi: 10.1186/s13578-023-01175-4 (PMC10693086; doi:10.1186/s13578-023-01175-4)

**Figure S1**

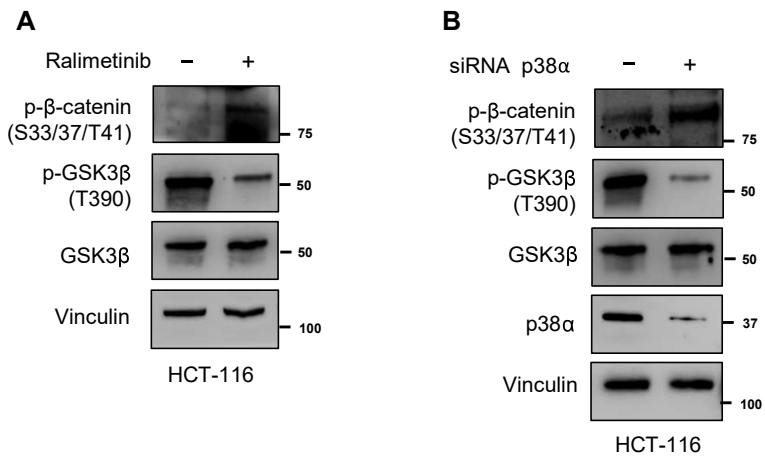

**Figure S2**

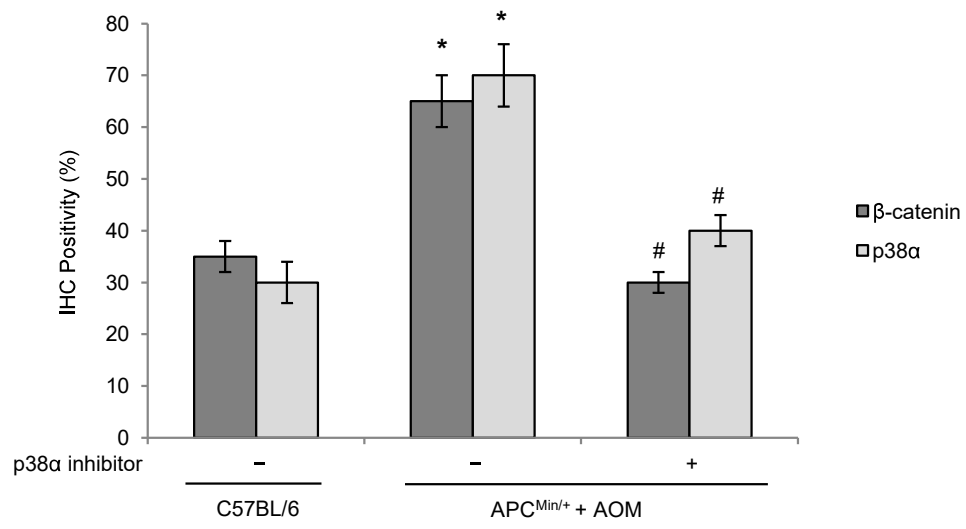

**Figure S3**

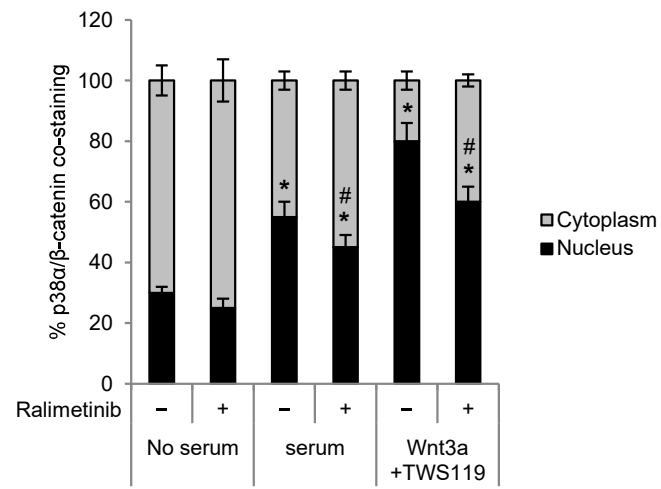

**Figure S4**

**A**

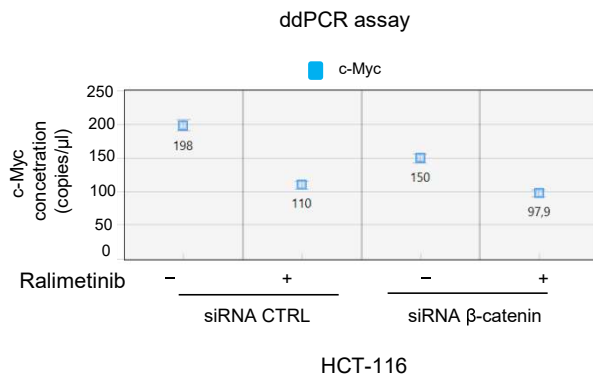

**B**

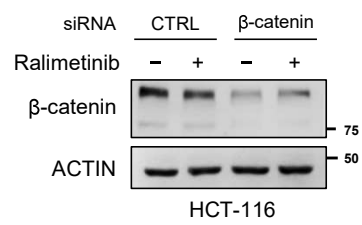

Supplement: Supplementary file 1 — Additional file 1: Figure S1. p38α pharmacological inhibition or genetic ablation modulates p-GSK3-β levels. (A-B) Immunoblotting analysis of p-β-catenin (S33/37/T41) (phospho-degradation signal) and p-GSK3β (T390) (phospho-inhibition signal) in HCT-116 CRC cells treated or not with the p38α inhibitor ralimetinib (10 μM) for 48 h (A) or silenced by genetic ablation for p38α (B). CTRL = Control. Results are representative of at least three independent experiments. Figure S2. Immunohistochemistry quantification of p38α and β-catenin protein expression in a CRC mouse model. Immunohistochemistry quantification of p38α and β-catenin protein expression in colon tissue from C57BL/6 and AOM-APCMin/+ mice treated as indicated in Fig. 2. Statistical analysis was performed using Student’s t-test: *P < 0.05 vs C57BL/6; #P < 0.05 vs. DMSO. Figure S3. Quantification of p38α and β-catenin immunofluorescence data. Quantification of the immunofluorescence analysis of p38α and β-catenin co-staining experiments shown in Fig. 4. Statistical analysis was performed using Student’s t-test: *P < 0.05 vs No serum; #P < 0.05 vs. DMSO. Figure S4. Analysis of β-catenin genetic ablation efficiency. (A) Quantification results of the digital droplet PCR (ddPCR) assay (copies/µL) of c-Myc mRNA expression as processed by QuantaSoft. HCT-116 CRC cells were silenced by genetic ablation for β-catenin and then treated or not with the p38α inhibitor ralimetinib (10 μM) for 24 h. The error bars represent the maximum and minimum Poisson distribution for the 95% confidence interval generated by QuantaSoft. (B) Immunoblotting analysis of c-Myc protein amount in HCT-116 CRC cells treated as in A. β-actin was used as a loading control. Results are representative of at least three independent experiments. [file 13578_2023_1175_MOESM1_ESM.pdf]
